# Integration of Catholic Values and Professional Obligations in the Provision of Family Planning Services: A Qualitative Study

**DOI:** 10.1001/jamanetworkopen.2020.20297

**Published:** 2020-10-12

**Authors:** Angela Marchin, Rebecca Seale, Jeanelle Sheeder, Stephanie Teal, Maryam Guiahi

**Affiliations:** 1 Department of Obstetrics and Gynecology, University of Colorado School of Medicine, Aurora; 2 Now with MEDNAX Health Solutions Partner, Sunrise, Florida; 3 Now with Planned Parenthood of the Rocky Mountains, Denver, Colorado; 4 Now with Planned Parenthood of the California Central Coast, Santa Barbara, California

## Abstract

**Importance:**

Religious leaders of the Catholic church created guidelines for practicing medicine, that involve reproductive care restrictions that may conflict with professional obligations.

**Objective:**

To explore how Catholic obstetrician-gynecologists integrate their religious values and professional obligations related to family planning services.

**Design, Setting, and Participants:**

In this qualitative investigation, in 2018, US-based obstetrician-gynecologists were recruited through an online survey and were invited to participate in audio-recorded telephone interviews using a semistructured interview guide. Participants were obstetrician-gynecologists who self-identified as Catholic and reported providing reproductive health care as follows: (1) provide natural family planning only (low practitioners), (2) provide additional contraceptive methods (moderate practitioners), and (3) provide family planning services including abortion (high practitioners). The study purposively sampled those with higher self-reported religiosity. Data were analyzed from November 2018 to February 2019.

**Main Outcomes and Measures:**

The primary outcome was understanding how participants describe integration of Catholic values with family planning service provision. The telephone interviews explored their integration of Catholic values and professional obligations, and 3 coders analyzed the responses using grounded theory.

**Results:**

Among the 34 Catholic obstetrician-gynecologists interviewed (27 women [79.4%]), there were 10 low, 15 moderate, and 9 high practitioners from 19 states. Participants’ description of morality was consistent with Albert Bandura’s Social-Cognitive Theory of Moral Thought and Action. The findings were used to create a modified framework. Within each group of physicians, 3 themes demonstrating their reconciliations between Catholic values and professional obligations emerged; each of these themes reflected one of the medical ethical principles of autonomy, beneficence, nonmaleficence, or justice. All 10 low practitioners primarily promoted natural family planning approaches to avoid iatrogenic risks and none provided abortion, reflecting nonmaleficence. Alternatively, moderate practitioners focused on nonmaleficence by offering contraception to prevent abortions. High practitioners primarily promoted patient autonomy by separating religious doctrine from medical practice. All had concerns for beneficence. In each group, 1 of the 4 medical ethical principles was underrepresented.

**Conclusions and Relevance:**

In this qualitative analysis, Catholic obstetrician-gynecologists establish their family planning care provision practices by emphasizing certain moral and/or ethical principles over others. These findings highlight how physician morality in the realm of family planning service provision often involves certain religious and/or professional reconciliations. Understanding the dilemmas Catholic obstetrician-gynecologists face can guide professional development efforts and inform ongoing discussions about conscientious objection and provision.

## Key Points

**Question:**

How do Catholic obstetrician-gynecologists integrate their religious beliefs with family planning services?

**Findings:**

In this qualitative study, 34 Catholic obstetrician-gynecologists were interviewed and demonstrated that their morality is developed and operationalized in a manner consistent with the Social-Cognitive Theory of Moral Thought and Action. Catholic values and professional obligations were differentially integrated and contributed to various practice patterns, ranging from provision of natural family planning only to abortion, and certain medical ethical principles were emphasized over others across these practice patterns.

**Meaning:**

These findings suggest that physician morality among Catholic obstetrician-gynecologists is not uniform and involves varying reconciliations with respect to religious and professional expectations.

## Teaser

This qualitative study explores how Catholic obstetrician-gynecologists integrate their religious values and professional obligations related to family planning services.

## Introduction

The intersection of family planning and Catholicism highlights a complex relationship between religion and medicine. The US Conference of Catholic Bishops provides medical guidelines called the *Ethical and Religious Directives for Catholic Health Care Services* (referred to hereafter as the Directives), updated in 2018.^1^ The Directives are intended for all practitioners in Catholic health settings and also for Catholic practitioners in non-Catholic settings.^1^ With respect to family planning, the Directives only allow natural family planning counseling for heterosexual married couples.^2^

A survey performed between 2008 and 2009 of more 1000 US obstetrician-gynecologists found that 7% did not provide 1 or more contraceptives,^3^ and 86% did not perform abortions.^4^ Although Catholicism was associated with nonprovision,^3,4^ many Catholic obstetrician-gynecologists provided family planning services.^3,4,5^ Given that many Catholic obstetrician-gynecologists do not strictly adhere to the Directives, we used qualitative methods to explore their integration of religious beliefs and professional obligations related to family planning services.

## Method

This qualitative study received exemption from the Colorado Multiple Institutional Review Board because it posed minimal risk to the participants. Informed consent was obtained as described later in this section. This study follows the Standards for Reporting Qualitative Research (SRQR) reporting guideline.^6^

Using an online REDCap^7^ survey, we recruited US-based obstetrician-gynecologists who self-identified as Catholic and queried about demographic characteristics, practice settings, self-identified religiosity, and family planning services provided; participants received a $5 gift card. On the basis of their responses, we sought Catholic obstetrician-gynecologists with a range of practice patterns and were interested in interviewing 3 clinically relevant groups, defined as low if they only provided natural family planning, moderate if they provided additional contraceptives, and high if they provided abortion routinely. We focused recruitment of low practitioners using contact information available on the American Association of Pro-Life Obstetrician-Gynecologists website.^8^ To recruit high practitioners, we emailed the Fellowship in Family Planning listserv. We also posted a survey link to Catholic Physicians and OB-GYN Moms groups on Facebook.

We recruited survey participants and purposively sampled the 3 practice groups for qualitative telephone interviews focusing on respondents with higher self-reported religiosity measures (very religious or moderately religious). To improve sample diversity, we also purposively recruited those who identified as male, non-White, and/or Hispanic-Latino. We excluded physicians for whom reproductive health care is not within their usual practice and trainees.

Before starting telephone interviews, we obtained verbal consent for participation and audio recording. Participants were informed that they could discontinue at any time or decline to respond and that all responses, including any quotations, would be deidentified. We then used a semistructured interview guide focused on the following: (1) importance of religion in professional and personal settings, (2) religion-related professional and personal conflicts, and (3) strategies used when experiencing conflict. Participants received a $100 gift card.

We conducted interviews until theoretical saturation^9^ occurred within each group from June through October 2018. All interview recordings were securely uploaded, professionally transcribed, and deidentified before analysis. We applied grounded theory^10,11,12^ to understand the phenomenon of how Catholic obstetrician-gynecologists integrate their religious beliefs and professional obligations as this previously lacked an applicable theory. The 3 coders (A.M., R.S., and M.G.) all had prior qualitative research experience and/or formal training. We initiated analysis by individually performing open-line coding of the first 6 interviews (2 from each group) and then met to reach consensus and begin a code dictionary. Using an iterative process, A.M. and R.S. individually first-line coded the remaining interviews, updated the code dictionary, and applied new codes to previously analyzed interviews. The 3 coders met at regular intervals to discuss memoranda and to perform second-line coding using Atlas TI software version 8 (Scientific Software Development GmbH) to formulate concepts, build categories, and create conceptual frameworks; data analysis continued from November 2018 to February 2019. To improve reliability, we used in-person group consensus to resolve differences and discussed each coder’s implicit biases throughout.

## Results

### Participants

We received survey responses from 174 obstetrician-gynecologists; 162 were eligible for interviews. We extended rolling interview invitations to 60 respondents. The final sample consisted of 34 physicians, including 10 low, 15 moderate, and 9 high practitioners from 19 states. Table 1 demonstrates participant characteristics; most were White (29 participants [85.3%]), female (27 participants [79.4%]), and non-Hispanic (29 participants [85.3%]); identified as very or moderately religious (29 participants [85.3%]); and attended church at least once per week (18 participants [52.9%]). There were more men in the low group (4 participants [40.0%]) compared with the moderate (2 participants [13.3%]) and high (0 participants) groups. As practitioners reported greater family planning service provision, it became difficult to purposively sample for higher self-identified religiosity.

**Table 1.** Participant Characteristics

Characteristic	Participants, No. (%)			
	Total (N = 34)	Provision Level		
		Low (n = 10)	Moderate (n = 15)	High (n = 9)
Gender				
Female	27 (79.4)	6 (60.0)	13 (86.7)	8 (88.9)
Male	6 (17.6)	4 (40.0)	2 (13.3)	0
Other	1 (2.9)	0	0	1 (11.1)
Age, y				
30-39	16 (47.1)	3 (30.0)	9 (60.0)	4 (44.4)
40-49	8 (23.5)	1 (10.0)	3 (20.0)	4 (44.4)
50-59	9 (26.5)	6 (60.0)	2 (13.3)	1 (11.1)
≥60	1 (2.9)	0	1 (6.7)	0
Race				
White	29 (85.3)	9 (90.0)	13 (86.7)	7 (77.8)
Black or African American	2 (5.9)	0	1 (6.7)	1 (11.1)
Asian	2 (5.9)	0	1 (6.7)	1 (11.1)
Multiple races	1 (2.9)	1 (10.0)	0	0
Hispanic or Latino ethnicity (n = 33)	5 (14.7)	2 (20.0)	2 (13.3)	1 (11.1)
Region of practice				
Midwest	12 (35.3)	4 (40.0)	5 (33.3)	3 (33.3)
South	9 (26.5)	3 (30.0)	4 (26.7)	2 (22.2)
Northeast	7 (20.6)	2 (20.0)	4 (26.7)	4 (44.4)
West	6 (17.6)	3 (30.0)	3 (20.0)	0
Practice setting				
Nonreligious facility	28 (82.4)	8 (80.0)	12 (80.0)	9 (100.0)
Catholic facility	7 (20.6)	2 (20.0)	5 (33.3)	0
Other religious facility^a^	2 (5.9)	0	2 (13.3)	0
Religiosity				
Slightly religious	5 (14.7)	0	1 (6.7)	4 (44.4)
Moderately religious	17 (50.0)	2 (20.0)	10 (66.7)	5 (55.6)
Very religious	12 (35.3)	8 (80.0)	4 (26.7)	0
Frequency of attendance at religious services				
Several times a week	4 (11.8)	4 (40.0)	0	0
Every week	14 (41.2)	5 (50.0)	9 (60.0)	0
2-3 times a month	3 (8.8)	0	3 (20.0)	0
About once a month	4 (11.8)	0	0	4 (44.4)
Several times a year	5 (14.7)	0	2 (13.3)	3 (33.3)
About once or twice a year	3 (8.8)	0	1 (6.7)	2 (22.2)
Missing	1 (2.9)	1 (10.0)	0	0
Patient services				
Natural family planning	30 (88.2)	10 (100.0)	12 (60.0)	8 (88.9)
Barrier method	23 (67.6)	0	14 (93.3)	9 (100.0)
Short-acting reversible contraception	24 (70.6)	1 (10.0)	14 (93.3)	9 (100.0)
Injection	24 (70.6)	0	15 (100.0)	9 (100.0)
Long-acting reversible contraception	24 (70.6)	1 (10.0)	14 (93.3)	9 (100.0)
Sterilization	24 (70.6)	0	15 (100.0)	9 (100.0)
Abortion	12 (35.3)	0	4 (26.7)	8 (88.9)

^a^ Includes Baptist and Seventh Day Adventist hospitals.

### Catholic Obstetrician-Gynecologist Morality

**Figure 1.**
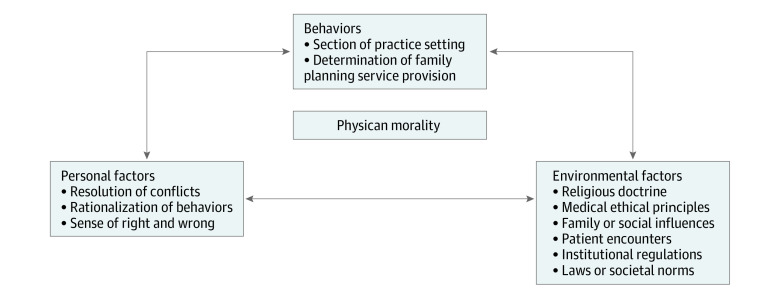
Modified Social Cognitive Theory of Moral Thought and Action Findings of Catholic obstetrician-gynecologists’ morality development organized by categories defined in Bandura’s Social Cognitive Theory of Moral Thought and Action.

Emerging themes demonstrated that Catholic religion was only one piece of a broader morality that influenced family planning practice decisions, consistent with Bandura’s Social-Cognitive Theory of Moral Thought and Action.^13^ We applied this theory, which states that morality is influenced by bidirectional interactions between external, personal, and behavioral factors, to organize the specific influences of Catholic obstetrician-gynecologists’ morality development and modified the framework, as demonstrated in Figure 1.

#### External Factors

The most common external factor cited by low practitioners cited was religious doctrine as they believed they had a moral imperative to adhere to church teachings. One stated, “To serve God is the most important thing in my life coming before even family and medicine.” Other physicians discussed societal norms in relation to beliefs that their patients did not generally need abortion services or fears of being harmed for providing abortion.

All participants discussed family and social influences. A moderate participant said, “I guess that’s what changed my thinking about contraception, is after I got married, actually, considering her [my wife’s] feelings.” Participants reflected on influences not necessarily specific to medical training.

Many moderate and high practitioners felt compelled to practice contrary to Catholic guidance on the basis of patient encounters. One high practitioner, explained, “…it’s easy to be ‘judgey’ when you think in absolutes, when you aren’t on that journey with the patient, and experiencing those times of the grayness of situations.” Those who worked in Catholic institutions often discussed how institutional regulations restricted medically necessary care. A moderate practitioner working in a Catholic Hospital stated, “…I really can’t offer my patient a lot of different options at our site…it kind of put me at odds with my religion.” Such physicians expressed greater tensions with their religion. The idea that the church should modernize to reflect the changing medical culture emerged as a nondominant theme, expressed by this moderate practitioner, “…the patriarchal or paternalistic nature of the church, it needs to evolve to help women and their providers deal with these certain real issues*.*” The intersection of medical ethical principles is described later.

#### Personal Factors

All participants used various coping strategies, including avoidance, talking to priests or therapists, and community support. Some described ongoing internal conflicts, such as this moderate practitioner: “I don’t want to turn my back on my patients, but I feel very guilty when I do perform that procedure [abortion].” This internal conflict led her to limit her abortion provision.

There was ubiquitous behavior rationalization. One of the low practitioners justified her practice as follows: “I had a senior resident who was like, ‘You’re denying care’…and I was like, okay, I think that this patient has plenty of resources.” Other participants believed the Catholic church could not be an absolute moral guide because of their observations of hypocrisy within the church, specifically regarding scandals and perceived inconsistencies between the church’s commitment to social justice and its restrictions on family planning.

There was frequent discussion of one’s internal sense of right and wrong, often referred to as *conscience*. Participants often stated they had innate senses that guided their provision of family planning services. Examples included, *“*I know I’m doing the right thing and in the end that’s all I can really be comfortable doing is trying to do what God wants me to do” (low practitioner), “I don’t think God thinks that you’re a bad person for giving contraception” (moderate practitioner), and “I’ve maintained this kind of balance, that I think that what I’m doing is consistent with Catholic teachings. I know others obviously would differ with that, but I’m at peace with where I am” (high practitioner).

#### Behaviors

Many described seeking job settings according to their family planning service approach, which often reinforced that their way of practicing was morally acceptable. A high practitioner stated, “Everyone in the practice practices the same as far as offering abortion services…we’re all very supportive of each other.” Many low practitioners discussed how their expertise in natural family planning provided a unique service that was well-received by patients and colleagues.

### Integration of Medical Ethics and Catholic Values in the Provision of Family Planning

**Figure 2.**
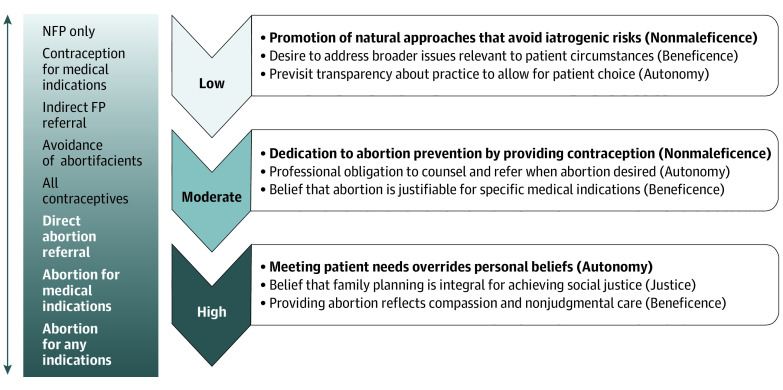
Integration of Medical Ethics and Catholic Values in the Provision of Family Planning Services Themes related to medical ethical principles in the context of Catholic influences and practice pattern. Primary themes are highlighted in bold.

We created a second framework that demonstrates the ethical and religious influences that guide family planning service patterns (Figure 2), because these were the primary influences expressed by participants. As themes emerged among the 3 provision groups, we noted that many reflected medical ethical principles and subsequently categorized themes as nonmaleficence, beneficence, autonomy, or justice^14^ (Figure 2). Representative quotations are reported in Table 2.

**Table 2.** Quotations Related to Provision of Family Planning Services Based on Practice Pattern

Theme	Quotation
Low	
Promotion of natural approaches that avoid iatrogenic risks (nonmaleficence)^a^	“…in the realm of family planning I have never really felt that I could justify introducing harm if the only thing I’m getting from it is the disruption of something that’s working normally to begin with…”
Desire to address broader issues relevant to patient circumstances (beneficence)	“Well, it was a thought that we weren’t addressing the base problem, which is that they were searching for something by having all these sexual partners…But we were just kind of putting a Band-Aid on that. Oh, but just don’t get pregnant without meaning to and that’ll be fine.”
Previsit transparency about practice to allow for patient choice (autonomy)	“…we really try and inform patients, like, this is our approach to practice, we know you have many choices, so we want you to understand this before you come here for care.”
Moderate	
Dedication to abortion prevention by providing contraception (nonmaleficence)^a^	“I have no problems prescribing birth control or doing tubal ligations. I know the Catholic church frowns upon birth control as well as tubals, but it’s something I feel like I’m doing less harm than more harm. If it were an ideal world I wouldn’t have to prescribe birth control to teenagers, or people who are single, but unfortunately it’s not. So I see it as the lesser of two evils. The other evil would be getting pregnant and then ending up with going to terminate the pregnancy.”
Professional obligation to counsel and refer when abortion desired (autonomy)	“I do counsel them on their options, and I do give them referrals, even though technically that goes against Catholic teaching as well, but I feel like as a physician I can’t make that decision for them, and I don’t want to put barriers in the way of them making that decision. I just don’t want to participate in the end result.”
Belief that abortion is justifiable for specific medical indications (beneficence)	“I was baptized when I was 10 days old, so I’ve never really known anything different, and that’s always been, “We’re pro life, we’re pro life, we’re pro life, we’re pro life.” But then when you talk to women and you see them making these tough decisions or they don't have the support systems, and just realizing that yeah this is a...they did this for their other children, or they did this because they knew they couldn’t do the best for that baby. I mean, it’s just like you can’t help but feel sympathy for them.”
High	
Meeting patient needs overrides personal beliefs (autonomy)^a^	“I think of what I do in a...non-religious way, for the most part. Like my religion is sort of...different from my work. So I suppose I compartmentalize my life.”
Belief that family planning is integral for achieving social justice (justice)	“What Jesus taught, washing the feet of people who are beggars. You take the lowest person and you treat them with compassion. Not that women getting abortions are low, I don’t mean that, but I mean in the sense that other people will reject them and treat them poorly, and we do the opposite. We try to mentor them and treat them well, which it’s a reversal, and I think it’s completely in line with the Catholic faith. Just looking out for the poor and the people who are rejected by others.”
Providing abortion reflects compassion and nonjudgment (beneficence)	“But I think just kind of using the basic fundamentals of Christianity which is just being kind to each other and just not judging people and not casting stones on other people until you’ve sort of been through that same situation.”

^a^ Indicates the dominant theme within each group.

Although we purposively sampled the participants into 3 groups on the basis of survey responses, we found variation in service provision within the low and moderate groups, as demonstrated by the spectrum of services delineated adjacent to the provision levels in Figure 2. Some of the low practitioners provided contraception for medical indications and/or suggested other service practitioners without necessarily providing direct referrals. The moderates varied more, with some avoiding contraceptives that the Church states are abortifacients, and others providing abortion in specific circumstances.

#### Low Provision

The most prominent theme among low practitioners was their promotion of natural approaches to avoid iatrogenic risks, classified as nonmaleficence. One said, “So for me to say I’m not gonna do contraception, that’s really not fair to the patient, not to give them a different alternative.” These participants promoted natural family planning as an effective method without harmful adverse effects.

The low provision group addressed beneficence based on their desire to address broader patient issues. Multiple participants called contraception a Band-Aid that other obstetrician-gynecologists used to avoid dealing with underlying physical or social issues. Often, they described their role as a physician as broader: “I think we are called to try to take care of some of that spiritual component also.”

Participants also described dedication to previsit transparency about their practices to support patient autonomy. For example, one said, “We’re not keeping you from that [birth control], we’re just telling you right up front, because we would never want to deceive.” These participants avoided conflicts by encouraging patients to seek care elsewhere when patient requests did not align with their values.

#### Moderate Provision

The primary theme among the moderate practitioners was their dedication to abortion prevention by providing contraception. One stated, “…there’s a huge difference between preventing pregnancy and killing a baby once it’s conceived.” These practitioners acknowledged that providing contraception was against Catholic doctrine, but saw this as “the lesser of two evils.” We categorized this as nonmaleficence on the basis of their concern for fetal harm.

The moderate group expressed respect for patient autonomy by acknowledging their obligation to refer for abortion when desired by patients, irrespective of their own beliefs. One stated, “I strive to not let them know what I’m personally thinking about that [abortion] and I provide them with resources so that they can get the care that they’re needing.” Many reiterated their attempts to conceal their opinions of abortion to avoid alienating patients.

Most moderate practitioners believed that abortion is justifiable for specific medical indications, such as fetal anomalies, reflecting beneficence. In these circumstances, physicians expressed greater empathy for their patients and some were comfortable performing the abortion. Another stated, “…that’s kind of been my way of getting through those situations, that this is for the mother and also that it was an extremely poor outcome if the pregnancy were to continue.” In such circumstances, they often felt abortion was in the best interest of their patients.

#### High Provision

The most prominent theme among the high practitioners was their belief that meeting patient needs overrides their personal beliefs, classified as autonomy. Many expressed how they often compartmentalize their religion and their profession, but also commented how Catholicism motivated their respect for persons. One participant said: “…when I pull upon how religion may have informed or impacted my relationship to reproductive health…it relates to a compassionate desire to respect women’s human integrity and autonomy.”

The high practitioners were the only ones who emphasized justice, believing that family planning is integral for achieving social justice. This was motivated by Catholic principles as described here: “…my Catholic upbringing and the exposure that I’ve had to Catholicism has made me very aware of social justice issues…and as part of that, I really believe that family planning is actually part of social justice.” Many commented on the responsibility to provide a service that is difficult to access.

High practitioners also expressed how providing abortion reflects compassionate and nonjudgmental care, classified as beneficence. One described the harmonious relationship between her religion and abortion provision: “…what I do is completely in line with the important part of Catholicism…we’re just taking care of people who need help, people who are in distress, people who are underserved, people who need empathy and compassion.” The high practitioners linked their Catholic faith with the need to provide family planning services.

## Discussion

Our study describes how moral and ethical values are integrated in the context of Catholicism and medical practice. We found that Catholic obstetrician-gynecologist morality was developed and operationalized in a manner consistent with the Social-Cognitive Theory of Moral Thought and Action.^13^ Morality among Catholic obstetrician-gynecologists was not uniform and involved varying reconciliations with respect to religious and professional expectations, with certain religious or ethical principles emphasized over others.

Our finding that Catholic physician morality was shaped by multiple interacting factors is consistent with the experiences of Catholic patients.^15,16^ The church’s approach to family planning is based on *Humanae Vitae*, an encyclical from Pope Paul VI in 1968 that established that sexual intercourse must have both “love-giving” and “life-giving” intentions.^17^ Participants discussed this as an ideal, but also expressed that Catholic values such as compassion, caring for the underserved, and social responsibility may allow for broader acceptability of family planning services. Notably, no group emphasized all 4 medical ethical principles. This likely highlights that with respect to religion and reproductive care, moral and/or ethical compromises must be made. Most prominently, the low and moderate practitioners emphasized the principle of nonmaleficence, the high practitioners emphasized autonomy and were the only group to underscore justice, and all commented on beneficence. Nonmaleficence and beneficence are the oldest among the medical ethical principles, originating in the time of Hippocrates. The concepts of autonomy and justice are newer concepts that indicate a shift in the practice of medicine to one where a patient’s personal agency surpasses the physician’s professional opinion.^18,19^ Some participants expressed that the Catholic church should modernize to reflect the changing medical culture.

Concerns that health professionals may compromise their moral values if forced to provide certain services have led to conscientious objection protections. Recently, the US Department of Health and Human Services attempted to strengthen such protections, issuing a rule prohibiting discrimination against individuals and health care institutions who act according to their consciences.^20^ Low and some moderate practitioners identified how conscientious objection is an important mechanism to resolve conflicts between personal and professional values. On the other hand, some moderate and many high practitioners expressed how provision of services was a common way of achieving a clear conscience. Conscientious provision has been previously described among abortion practitioners; that is, their conscience is what informs abortion provision.^21,22^ Those who worked in Catholic settings discussed family planning restrictions, particularly restrictions on services deemed medically necessary, as a source of conflict with their religion; such concerns have been highlighted in other investigations of physicians in Catholic settings.^23^ These findings suggest that without conscientious provision protections, many physicians may face unresolved moral conflicts when faced with organizational restrictions at the institutional or governmental levels.

### Limitations and Strengths

This study has limitations that should be addressed. We limited our recruitment to obstetrician-gynecologists and cannot comment on other physicians. Both our study population and research team were racially and ethnically homogeneous, so the experiences and interpretations may not reflect underrepresented populations. Furthermore, a higher proportion of the low practitioners were men compared with the other 2 groups; thus, it is possible we were unable to detect the association of gender differences with moral development. Focusing on only 1 religion with clear guidelines for family planning service provision was a strength, because it allowed for nuanced interpretations. Although we used convenience sampling, we had a robust response to our recruitment survey, allowing us to purposively sample and reach theoretical saturation within each group. To enhance reliability and validity, multiple coders analyzed the data while maintaining reflexivity.

## Conclusions

These findings provide guidance for effective interventions. Given the benefits seen from values clarification exercises, and a Providers Share workshop,^24,25^ Catholic physicians will likely benefit from similar workshops tailored to the inherent conflicts and the sensitive nature of this intersection of medicine and religion. Understanding the dilemmas faced by Catholic obstetrician-gynecologists and their moral and ethical decision-making can improve discourse on this topic and help destigmatize the varying choices Catholic patients and health professionals make. Physician concerns may provide insight to church leaders charged with providing guidance for modern medical issues. As an example, the low practitioners highlighted the need for transparency about services to support patient autonomy, yet prior studies have suggested that many Catholic institutions appear to limit transparency about such restrictions to care.^2,26,27^ In addition, although interpretation of certain Catholic teachings are seemingly inconsistent with family planning service provision, our participants highlighted how other Catholic values are consistent with provision. Broader consideration of these values and Catholic teachings such as the Principle of Toleration may provide a mechanism for more permissive reproductive care guidelines rooted in religious values.
